# Metallothionein Isoforms in Acute Myeloid Leukemia: Roles in Survival, Differentiation, and Stress Adaptation

**DOI:** 10.3390/hematolrep18050062

**Published:** 2026-08-31

**Authors:** Shinichiro Takahashi

**Affiliations:** 1 Division of Laboratory Medicine, Faculty of Medicine, Tohoku Medical and Pharmaceutical University, 1-15-1 Fukumuro, Miyagino-ku, Sendai 983-8536, Miyagi, Japan; shintakahashi@tohoku-mpu.ac.jp; Tel.: +81-22-290-8889; 2 Institute of Molecular Biomembrane and Glycobiology, Tohoku Medical and Pharmaceutical University, 4-4-1 Komatsushima, Aoba-ku, Sendai 981-8558, Miyagi, Japan

**Keywords:** metallothioneins, acute myeloid leukemia, isoform-specific regulation, drug resistance, stress adaptation, redox homeostasis, epigenetic regulation

## Abstract

Metallothioneins (MTs) are cysteine-rich metal-binding proteins traditionally recognized for their roles in metal homeostasis and antioxidant defense. Recent studies have revealed broader and functionally diverse roles of MT isoforms in acute myeloid leukemia (AML). This review summarizes current evidence regarding MT-mediated regulation of leukemic cell survival, differentiation, and therapy-associated stress adaptation. MT1 family members, particularly MT1X and MT1G, have been associated with proliferation, chemoresistance, and differentiation blockade in specific experimental contexts, whereas MT2A and MT3 exhibit tumor-suppressive effects in selected AML models. MT isoforms also participate in oxidative stress adaptation and redox regulation, including responses to azacitidine, doxorubicin, and ferroptotic stress. However, the available evidence is derived largely from cell-line studies, selected molecular subtypes, transcriptomic analyses, and limited primary-patient datasets. Accordingly, isoform-specific MT functions should be interpreted as context-dependent rather than uniformly applicable across AML. Further validation in independent primary AML cohorts and genetically defined subtypes is required to establish their clinical utility as biomarkers or therapeutic targets.

## 1. Introduction

Metallothioneins (MTs) are multifunctional cysteine-rich proteins that regulate intracellular metal homeostasis and protect cells against oxidative and electrophilic stress. In my previous review published in 2012, I summarized the canonical molecular functions of MTs, emphasizing their roles in zinc buffering, free radical scavenging, anti-apoptotic signaling, and drug resistance, while also outlining the limited evidence then available regarding MT expression in hematological malignancies [1]. At that time, MTs in leukemia and lymphoma were largely interpreted within the classical framework of cytoprotection against oxidative injury and chemotherapy-induced apoptosis.

However, over the past decade, the understanding of MT biology in hematologic malignancies has evolved toward a more dynamic view in which individual MT isoforms may participate in leukemic cell adaptation and disease progression. Accumulating evidence over the past decade has shown that MT1 family members are predominantly associated with pro-leukemic functions, including proliferation, survival, differentiation blockade, and drug resistance [2,3,4,5,6], whereas MT2A and MT3 have been reported to exhibit tumor-suppressive effects [7,8] in specific contexts. In addition, MT isoforms have also been implicated in oxidative stress and therapy-associated responses, including azacitidine-resistant networks [9]. These observations support investigation of MTs as context-dependent modulators of AML biology, while emphasizing that the evidence varies by isoform, model, and molecular subtype. Differentiation blockade is now recognized as a central hallmark of AML. Beyond transcriptional regulation, dysregulation of chromatin and metabolic processes contributes to maintaining this undifferentiated state in AML [10]. Collectively, these findings support an updated framework in which MT is viewed not simply as a marker of oxidative defense, but as a functionally important mediator of leukemia cell survival, therapy resistance, and stress adaptation in hematologic malignancies. Since the publication of the previous review in 2012 [1], substantial advances have been made in understanding the roles of MT isoforms in hematological malignancies. This review provides an updated perspective by integrating recent findings on MT-mediated regulation of leukemic cell survival, differentiation, and therapy-associated stress adaptation in AML. 

## 2. Literature Search Strategy

This narrative review was updated through August 2026 using PubMed searches supplemented by reference-list screening of relevant original articles and reviews. Search terms included combinations of “metallothionein,” “MT1,” “MT1G,” “MT1X,” “MT2A,” “MT3,” “acute myeloid leukemia,” “AML,” “differentiation,” “oxidative stress,” “redox,” “ferroptosis,” and “drug resistance.” Priority was given to original studies evaluating MT isoforms in AML cell lines, primary AML samples, animal models, or transcriptomic/proteomic datasets. Studies outside AML were included only when they provided mechanistic context not yet established in AML. Because this article is a narrative rather than a systematic review, no formal meta-analytic or PRISMA-based selection process was applied; the revision instead emphasizes transparent distinction between experimentally supported AML findings, associative omics observations, and proposed mechanistic interpretations.

## 3. MT in AML Cell Survival and Therapy Response

Zhu et al. [11] revealed that multiple MTs, ZIP transporters (Zrt/Irt-like protein family; SLC39A), which mediate zinc influx into the cytosol, and ZnT transporters (zinc transporter family; SLC30A), which mediate zinc efflux or sequestration into intracellular compartments, are differentially expressed in AML, indicating that zinc homeostasis is broadly dysregulated in leukemic cells. In AML, zinc depletion alters the expression of most positively expressed MT isoforms, along with several zinc transporters, supporting the concept that MT-associated zinc homeostasis is functionally linked to leukemia cell survival and may represent a therapeutic vulnerability. Consistent with this concept, recent evidence demonstrated increased intracellular zinc levels and upregulation of zinc influx transporters in AML blasts, with ZIP10 targeting impairing zinc uptake and leukemic cell growth [12]. These studies provide transcriptomic, association-level evidence rather than functional validation of individual MT isoforms.

A recent study by Park and Miyano [9] demonstrated that network analysis of azacitidine-resistant AML cells identified the *MT* gene family as a central component of a drug-resistance-associated regulatory circuit, together with genes involved in adaptive stress signaling. These findings establish an association between MT-family upregulation and the resistant state, but they do not by themselves demonstrate that MT induction is a direct cause of azacitidine resistance.

Several groups have demonstrated the role of cell proliferation and survival in AML. Colom Diaz et al. [5] recently reported an important role of MT1 in leukemic cell survival. They revealed that MT1 isoforms are selectively upregulated in Dnmt3a;Npm1-mutant AML and function as critical survival factors that sustain leukemic proliferation, suppress differentiation- and oxidative stress-associated programs, and promote leukemogenicity in vivo. Genetic disruption of *MT1G* impairs AML cell growth in both murine and human Dnmt3a;Npm1-mutant models, identifying MT-dependent stress adaptation as a lineage-specific therapeutic vulnerability in this AML subtype. Consistently, we previously reported that MT1G overexpression accelerates G1/S cell-cycle transition in NB4 acute promyelocytic leukemia cells, as shown by increased S-phase entry and upregulation of cyclin E and cyclin A following serum stimulation. In addition to suppressing retinoic acid-induced differentiation, MT1G confers resistance to cytosine arabinoside (Ara-C), indicating that it promotes both proliferative adaptation and chemoresistance in leukemic cells [3].

Xin et al. [2] revealed that MT1X, a major MT isoform, is overexpressed in AML and functions as a biologically active driver of leukemic progression, promoting cell proliferation, survival, and resistance to doxorubicin through NF-κB-associated signaling. Its expression is negatively regulated by miR-376a-3p, the restoration of which suppresses AML cell growth, induces apoptosis, and reverses MT1X-mediated malignant phenotypes, identifying the miR-376a-3p/MT1X axis as a potential therapeutic target in AML.

In contrast, Pan et al. [7] reported that MT2A functions as a negative regulator of leukemic cell growth in HL60 AML cells: its overexpression suppresses proliferation, induces apoptosis, and causes G2/M cell-cycle arrest, whereas MT2A knockdown enhances proliferation and reduces apoptosis. Mechanistically, these effects are associated with inhibition of NF-κB signaling through increased IκB-α and reduced p-IκB-α and cyclin D1 expression, indicating that MT2A modulates AML cell survival via NF-κB-dependent pathways. Consistent with the tumor-suppressive role of MT isoforms, Tao et al. [8] reported that MT3 acts as a putative tumor suppressor in pediatric AML, where its expression is frequently silenced by promoter hypermethylation in both leukemia cell lines and primary patient samples. Restoration of MT3 expression suppresses AML cell proliferation and induces apoptosis, partly through the dysregulation of apoptosis-related genes, including *FOXO1*, suggesting that epigenetic loss of MT3 contributes to leukemogenesis.

**Figure 1:**
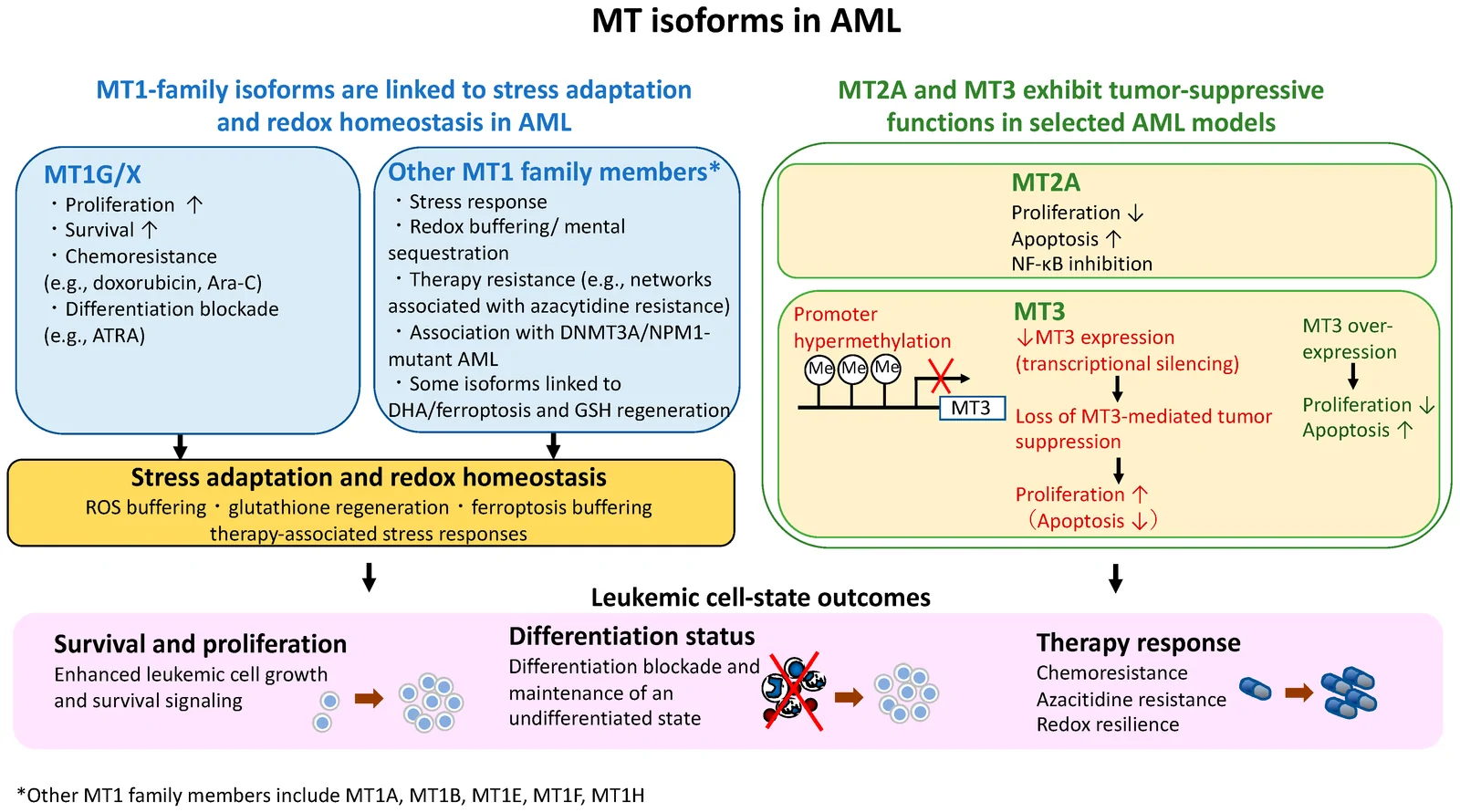
Proposed framework for isoform-specific MT functions in AML. MT1X, MT1G, and MT1-family findings are linked mainly to leukemic survival, differentiation blockade, therapy response, and stress-adaptive phenotypes in defined AML models. MT2A and MT3 show growth-suppressive effects in selected models. For *MT3*, promoter hypermethylation is associated with reduced MT3 expression, whereas experimental restoration of MT3 expression suppresses proliferation and promotes apoptosis. Arrows indicate the direction of the proposed functional relationships; upward and downward arrows indicate increases and decreases, respectively. Redox homeostasis and stress adaptation are presented as context-dependent functions supported in selected MT isoforms and experimental settings, rather than as universal properties of all MT isoforms.

Collectively, these studies indicate that MT isoforms play divergent and isoform-specific roles in AML biology rather than functioning as a uniform molecular group. While MT1 family members, including MT1X and MT1G, appear to promote leukemic survival, proliferation, and therapy resistance, other isoforms, such as MT2A and MT3, exhibit tumor-suppressive properties by inhibiting proliferation and promoting apoptosis. This functional heterogeneity suggests that the biological effects of MT in AML are highly context-dependent and may reflect differences in isoform-specific regulation, cellular localization, and interaction with distinct signaling pathways. The strength of evidence differs among isoforms, and several conclusions are based on single cell lines or selected molecular subsets. Figure 1 therefore presents these relationships as a proposed framework rather than a universal AML pathway, and Table 1 explicitly distinguishes experimental systems and evidence types.

## 4. MT in Differentiation Control

PU.1 is a key transcription factor for hematopoiesis, and its downregulation plays a pivotal role in AML pathogenesis [13,14]. To clarify the function of PU.1, we previously identified several MT isoforms (MT1G, MT1A, and MT1E) as direct target genes of PU.1 through epigenetic mechanisms involving promoter CpG methylation and MeCP2 recruitment [15]. In primary AML specimens, MT1 isoform expression is inversely correlated with PU.1 levels, indicating that loss of PU.1 derepresses *MT1* genes and may contribute to aberrant leukemic cell-state regulation. 

In NB4 acute promyelocytic leukemia cells, MT1G overexpression attenuated ATRA-induced differentiation, reduced G1 arrest and nitroblue tetrazolium positivity, and altered expression of myeloid differentiation-related genes [6]. Together with PU.1-regulation studies [15], these findings support a relationship between MT1G and differentiation control in this specific APL model. Evidence that the same mechanism operates across genetically diverse non-APL AML remains limited.

Importantly, independent studies provide both supportive and cautionary context for the proposed relationship between MT expression and hematopoietic differentiation. Abdel-Mageed et al. showed in K562 erythroid progenitor cells that MT-IIA overexpression inhibited erythropoietin- or sodium butyrate-induced differentiation, an effect that was partially reversed by an MT antisense oligonucleotide [16]. Consistent with an association between MT1G and impaired myeloid maturation, Ogushi et al. independently reported that cadmium exposure in HL-60 acute myelocytic leukemia cells increased MT1G expression while inhibiting the ATRA-induced increase in nitroblue tetrazolium reduction activity; however, the pleiotropic effects of cadmium preclude attribution of the differentiation defect specifically to MT1G [17]. Conversely, Maghdooni Bagheri et al. found higher MT isoform expression in more mature hematopoietic precursor cell lines than in an immature precursor line and observed increased MT transcription and protein expression during phorbol ester-induced K562 differentiation [18]. These independent findings indicate that MT expression can either accompany or oppose differentiation depending on isoform, lineage, and experimental stimulus, and therefore argue against a universal differentiation-blocking function of MTs.

Within AML, the most direct causal evidence for MT1G-mediated differentiation blockade remains the NB4/PU.1 studies [6,15]. The independent HL-60 study [17] provides convergent but non-causal evidence, whereas studies in K562 and other hematopoietic precursor models [16,18] demonstrate that the relationship between MT expression and differentiation is context-dependent. Validation in genetically diverse primary AML samples is therefore required.

Beyond AML, post-2012 studies in other cellular systems also support a broader link between MT isoforms and the control of differentiation. MT3 has been reported to promote osteoblast differentiation [19] and osteoclast differentiation [20], whereas MT1/MT2 [21] and MT3 [22] have been shown to modulate adipocyte differentiation in a context-dependent manner. Although these findings were obtained in non-hematologic models, they consistently indicate that MTs can influence lineage-specific differentiation programs across multiple cellular systems.

Taken together with observations in AML, including the inhibitory effect of MT1G on retinoic acid-induced myeloid differentiation [6], these findings support the view that MT isoforms may participate in the regulation of differentiation processes in a cell type- and isoform-dependent manner. 

## 5. MT Isoforms in the Context of AML Heterogeneity

AML comprises biologically distinct entities defined by differentiation state, cytogenetic abnormalities, and recurrent molecular lesions. The available MT literature does not yet support a uniform isoform expression pattern across these entities. The strongest subtype-specific evidence currently concerns DNMT3A/NPM1-mutant AML, in which an MT1-family program was identified as a survival dependency in murine and human models [5]. By contrast, MT1G differentiation studies were performed mainly in the NB4 acute promyelocytic leukemia model [3,6], MT2A was examined in HL60 cells [7], and MT3 silencing was reported in pediatric AML cell lines and primary samples [8]. These observations should not be generalized to FLT3-mutated, TP53-mutated, IDH1/2-mutated, core-binding-factor, or other AML subsets in the absence of direct validation. Public transcriptomic analyses indicate broad dysregulation of zinc-homeostasis genes in AML [11], but such associations do not establish isoform-specific causal dependencies within each molecular subtype.

## 6. Molecular and Redox Regulation of MT Isoforms in AML

The biological effects of MT isoforms in AML are shaped by multiple layers of regulation, including transcriptional, epigenetic, post-transcriptional, signaling, and redox mechanisms. However, the strength of evidence differs substantially among these regulatory levels, and many proposed connections remain incompletely validated in AML. MT1G, MT1A, and MT1E have been identified as PU.1-regulated genes, with promoter CpG methylation and MeCP2 recruitment contributing to their transcriptional control [15]. *MT1X* is post-transcriptionally regulated by miR-376a-3p and has been linked to NF-κB-associated survival signaling [2], whereas MT2A-mediated growth suppression in HL60 cells is associated with reduced NF-κB signaling [7]. *MT3* provides a distinct epigenetic example: promoter hypermethylation reduces its expression in pediatric AML, and restoration of MT3 suppresses proliferation and promotes apoptosis [8].

Redox regulation represents another functional layer of MT biology. During DHA-induced ferroptotic stress, MT expression is induced, inhibition of MT2A and MT1M enhances ferroptotic cell death, and MT-dependent antioxidant responses contribute to glutathione regeneration [4]. These experiments provide direct evidence that selected MT isoforms can buffer ferroptotic and oxidative stress in AML models. By contrast, enrichment of MT-family and metal-response genes in azacitidine-resistant cell-line networks [9] is associative and does not establish that MT induction itself causes drug resistance. Redox-dependent protein S-glutathionylation has also been linked to azacitidine sensitivity and resistance [23], further supporting the relevance of cellular redox state to therapy response, although a direct isoform-specific MT mechanism has not been established.

PI3K/AKT, MAPK, NRF2, and p53 pathways are established components of AML survival, stress signaling, and redox regulation [24,25,26]. Nevertheless, direct mechanistic links between these pathways and individual MT isoforms remain insufficiently defined in AML. Similarly, although mitochondrial ROS production is central to oxidative-stress biology and treatment response in AML, direct evidence linking specific MT isoforms to mitochondrial regulation is currently limited. Therefore, these pathways provide important mechanistic context and priorities for future investigation, rather than serving as components of an established universal MT signaling axis. Taken together, current evidence supports a model in which transcriptional and epigenetic control determines MT isoform expression, while selected MT isoforms can in turn modify survival signaling and redox buffering. The consequences of this regulation appear to depend on isoform, cellular context, and therapeutic stress.

## 7. Clinical and Therapeutic Implications

The translational potential of MT isoforms remains promising but preliminary. Expression analyses of zinc-homeostasis-related genes suggest possible prognostic associations in AML [11], and the selective MT1 dependency reported in DNMT3A/NPM1-mutant AML raises the possibility of genetically restricted therapeutic vulnerabilities [5,27]. Nevertheless, no MT isoform is currently validated for routine AML diagnosis, risk stratification, treatment selection, or response monitoring, and no isoform-specific MT inhibitor has an established clinical role in AML. Direct MT targeting is complicated by the high structural similarity among MT family members, their physiological roles in zinc and copper buffering, antioxidant defense, and tissue protection, as well as the possibility that systemic inhibition could disrupt normal metal homeostasis. More fundamentally, MTs are small, cysteine-rich, nonenzymatic proteins that lack conventional catalytic pockets, further limiting the development of selective small-molecule inhibitors. Targeting upstream regulatory pathways, including the miR-376a-3p/MT1X axis, PU.1-associated epigenetic machinery, or redox-buffering pathways, remains investigational. Moreover, systemic interference with metal-response or redox pathways could impair normal hematopoietic stem and progenitor cells and cause adverse effects in other organs. Isoform selectivity, molecular-subtype specificity, therapeutic windows, and effects on normal hematopoiesis must therefore be established before such approaches can be translated clinically. Prospective studies in molecularly annotated primary AML cohorts are required before MT expression can be considered a clinically actionable biomarker or therapeutic target.

## 8. Limitations and Future Directions

Several limitations should be emphasized. Many mechanistic observations derive from individual cell lines, including NB4 and HL60, or from selected preclinical models, limiting generalizability across AML. Cell-line and in silico models cannot fully reproduce the clonal heterogeneity, bone-marrow microenvironment, stromal and immune interactions, and treatment-driven evolution observed in primary AML. Transcriptomic and network analyses provide valuable associations but do not by themselves demonstrate causality. No de novo analysis of TCGA, GEO, or other public datasets was performed in this narrative review. Primary-patient validation and independent clinical cohorts remain limited for most individual MT isoforms. In addition, the term “MT1” is not always used consistently and may refer to multiple *MT1*-family genes or an MT1-associated expression program rather than a single isoform. Future work should integrate genetically annotated primary samples, single-cell and bulk transcriptomic/proteomic datasets, functional perturbation, and longitudinal clinical outcomes, with particular attention to FLT3-, NPM1-, TP53-, and IDH1/2-mutated and core-binding-factor AML. 

## 9. Discussion

Recent studies have expanded the role of MTs in AML beyond their classical function as passive antioxidant proteins. The available evidence, however, does not support a single uniform function for MTs across AML. Rather, MT isoforms appear to exert heterogeneous and context-dependent effects. MT1 family members, including MT1X and MT1G, have been associated with leukemic proliferation, survival, differentiation blockade, and treatment resistance in defined experimental settings, whereas MT2A and MT3 have shown growth-suppressive and pro-apoptotic effects in selected models. The selective dependence on an MT1-associated program, as reported in DNMT3A/NPM1-mutant AML, further suggests that MT function may also vary according to molecular subtype [5]. Thus, the apparently divergent functions of MT isoforms should be interpreted in relation to isoform identity, cellular context, molecular background, and experimental model rather than as a uniform property of the MT family.

Differentiation-related findings particularly illustrate this context dependence. MT1G overexpression suppresses ATRA-induced differentiation in NB4 cells [6], and independent hematopoietic studies provide both supportive and contrasting observations. MT-IIA overexpression inhibited erythropoietin- or sodium butyrate-induced differentiation in K562 cells [16], whereas cadmium exposure in HL-60 cells was associated with MT1G induction and reduced ATRA-induced differentiation [17], although the latter observation does not establish MT1G-specific causality. Conversely, increased MT expression has also been reported in more mature hematopoietic precursor cell lines and during induced differentiation [18]. Collectively, these findings argue against a universal differentiation-blocking function of MTs and instead suggest that the relationship between MT expression and differentiation depends on isoform, lineage, and stimulus.

Redox adaptation represents another important but similarly context-dependent aspect of MT biology in AML. MT induction during DHA-induced ferroptotic stress and enhancement of ferroptotic cell death following inhibition of selected MT isoforms provide direct experimental evidence that MTs can contribute to cytoprotective redox responses in AML models [4]. In contrast, enrichment of MT-family genes in azacitidine-resistant regulatory networks [9] represents an associative observation and does not establish that MT upregulation itself causes drug resistance. Together with findings involving doxorubicin and Ara-C responses, these studies suggest that MT-associated redox buffering may contribute to treatment adaptation in particular experimental settings, but current evidence does not establish a universal MT-mediated stress-adaptation pathway across AML.

From a translational perspective, MT isoforms may ultimately have biomarker or therapeutic relevance, but the available evidence remains preliminary. The selective MT1 dependency reported in DNMT3A/NPM1-mutant AML suggests a potential subtype-specific vulnerability [5], while differential expression of individual MT isoforms may reflect distinct biological states. However, no MT isoform is currently validated for AML risk stratification, therapeutic prediction, or treatment selection, and direct MT targeting is complicated by structural similarity among MT family members and their physiological roles in metal homeostasis and antioxidant defense. Future studies should integrate isoform-resolved analyses with molecularly annotated primary AML samples, functional perturbations, and longitudinal clinical outcomes. Such studies will be required to determine whether individual MT isoforms function as mechanistic drivers, context-dependent modifiers, or biomarkers of specific AML states and whether MT-associated pathways can be therapeutically exploited without disrupting physiological metal homeostasis.

## 10. Conclusions

MT isoforms are associated with distinct and context-dependent functions in AML, including leukemic survival, differentiation, and redox adaptation. Current evidence supports isoform- and model-specific roles, rather than a universal MT stress-adaptation program. Further validation in genetically defined primary AML cohorts is required to establish whether individual MT isoforms can serve as clinically useful biomarkers or therapeutic targets.

## Figures and Tables

**Table 1:** Isoform-specific roles of MTs in AML. Representative MT isoforms exhibit distinct and context-dependent biological roles in AML. Representative findings are summarized, along with the AML context, experimental system, evidence type, and potential clinical implications. Clinical applications remain investigational unless otherwise stated.

Isoform	AML Context/Model	Functional Category	Biological Role in AML	Mechanistic Axis	Evidence Type	Potential Clinical Implication	Reference
MT1X	AML, mainly cell-line studies	Oncogenic	Promotes proliferation, survival, and chemoresistance	NF-κB activation; miR-376a-3p axis	Experimental/preclinical	Doxorubicin resistance; therapeutic target candidate	Xin et al., 2022 [2]
MT1G	APL/NB4 cell-line studies	Pro-leukemic	Enhances cell cycle progression and blocks differentiation	Cyclin E/A induction; PU.1 repression	Experimental/preclinical	Ara-C resistance; differentiation blockade	Hirako et al., 2021 [3]; Hirako et al., 2014 [6]
MT1	DNMT3A/NPM1-mutant AML Murine/human AML models, expression analyses	Survival factor	Maintains leukemic cell survival in specific AML subtypes	Oxidative stress buffering; cell cycle support	Experimental/preclinical	DNMT3A/NPM1 AML vulnerability	Colom Diaz et al., 2026 [5]
MT2A	MT2A over-expressed HL60 cells	Tumor suppressor	Inhibits proliferation and induces apoptosis	NF-κB inhibition	Experimental/preclinical	Potential biomarker of favorable biology	Pan et al., 2021 [7]
MT3	Pediatric AML Cell lines + primary samples	Tumor suppressor	MT3 expression suppresses proliferation and promotes apoptosis; MT3 is frequently silenced in pediatric AML	Promoter hypermethylation; apoptosis-related genes including *FOXO1*	Experimental/epigenetic	Potential epigenetic relevance; clinical utility unvalidated	Tao et al., 2014 [8]
Multiple MT isoforms	DHA-ferroptosis model, Cell-line studies	Stress-adaptive	Buffer ferroptosis and oxidative stress	Glutathione regeneration; ferroptosis suppression	Experimental/preclinical	Resistance to oxidative therapies	Grignano et al., 2023 [4]
MT family	Azacitidine-sensitive/resistant AML, cell-line network analysis	Resistance-associated	Associated with therapy-resistance networks	Metal-response gene network (Zn/Cu/Cd)	Associative/network	Azacitidine-resistance association; causality unproven	Park et al., 2024 [9]
MT system	AML, Public transcriptomic/expression analyses	Prognostic regulator	Reflects AML survival and metabolic state	Zinc homeostasis dysregulation	Associative/omics	Prognostic biomarker potential	Zhu et al., 2023 [11]

## Data Availability

No new data were created or analyzed in this study. Data sharing is not applicable to this article.
